# Teledermatology and face-to-face pathways for basal cell carcinoma diagnosis in a southern European cohort: a comparative histopathologic analysis

**DOI:** 10.3389/fmed.2026.1713904

**Published:** 2026-02-02

**Authors:** Marta Cebolla-Verdugo, Carlos Llamas-Segura, Husein Husein-ElAhmed, Ricardo Ruiz-Villaverde

**Affiliations:** 1 Hospital Universitario San Cecilio, Servicio de Dermatología, Granada, Spain; 2 Hospital General de Baza, Baza, Spain

**Keywords:** teledermatology, prognosis, basal cell carcinoma, early diagnosis, skin cancer

## Abstract

**Background:**

Teledermatology has expanded markedly in the post-pandemic era; however, its impact on the histopathologic presentation of basal cell carcinoma (BCC) remains insufficiently characterized. Tumor depth and histologic subtype are key prognostic factors and may differ according to diagnostic pathway.

**Methods:**

We conducted a retrospective observational study at a tertiary referral hospital in southern Europe, including all histologically confirmed BCCs diagnosed in 2019 through face-to-face dermatology and in 2022 through teledermatology. A total of 486 tumors were analyzed (201 face-to-face; 285 teledermatology). Histologic subtypes (superficial, nodular, micronodular, sclerodermiform) were examined individually and grouped into non-aggressive (superficial/nodular) versus aggressive (micronodular/sclerodermiform) categories. Statistical analyses included Welch’s *t*-test, chi-square tests, and multivariable logistic regression.

**Results:**

Mean tumor depth was significantly lower in the teledermatology cohort (1.67 mm vs. 2.51 mm; *p* < 0.001). Histologic subtype distribution differed between pathways (χ^2^ = 41.3; *p* = 0.002): superficial BCCs were more frequent in face-to-face consultations (15.9% vs. 8.8%), whereas nodular and micronodular variants predominated in the teledermatology group (65.6% vs. 62.2 and 13.3% vs. 4.0%, respectively). Sclerodermiform tumors were slightly more common in face-to-face evaluations (17.9% vs. 12.3%). When regrouped into non-aggressive versus aggressive patterns, the distribution differed between pathways, although this categorization did not indicate a predominance of low-risk histology in the teledermatology cohort. In multivariable analysis, tumor depth was the only independent predictor of consultation pathway (*β* = −1.10; *p* < 0.001).

**Conclusion:**

Teledermatology was associated with the diagnosis of significantly shallower BCCs, even across subtypes, supporting its role in facilitating earlier histologic detection. Subtype differences were pathway-specific but did not fully account for the depth disparity. These findings highlight teledermatology as an effective diagnostic route for timely identification of BCC in contemporary clinical practice.

## Introduction

Basal cell carcinoma (BCC) is the most common skin cancer, accounting for approximately 80% of non-melanoma skin cancers worldwide (1). Its incidence continues to rise in Europe and globally, partly due to population aging and cumulative ultraviolet (UV) exposure (2, 3). Although BCC has a very low metastatic potential, its high prevalence and capacity for local tissue destruction represent a major public health burden (4). Early detection and treatment are crucial, as advanced cases may require mutilating surgery or, more recently, systemic therapy with Hedgehog pathway inhibitors, which are reserved for locally advanced or metastatic disease (5, 6).

Teledermatology has become increasingly integrated into dermatologic care over the past two decades, with its role further expanded during and after the COVID-19 pandemic. Multiple studies have demonstrated its utility for triaging skin lesions and improving access to specialist care. Previous research on teledermatology in skin cancer has primarily addressed diagnostic concordance, i.e., the agreement between the presumed clinical diagnosis made remotely and subsequent histopathological confirmation. Reported concordance rates are generally moderate to high, particularly when dermoscopy is incorporated into the teleconsultation process (7, 8).

Moreover, a recent systematic review (9) specifically addressing non-melanoma skin cancer confirmed that teledermatology achieves diagnostic accuracy comparable to face-to-face dermatology and emphasized its advantages in accessibility and reduced time-to-treatment (9). Nevertheless, no studies to date have evaluated whether consultation modality influences histopathological parameters of BCC, such as tumor depth or subtype distribution. Since these factors are strongly linked to prognosis, surgical complexity, and recurrence risk, their analysis in relation to diagnostic modality represents an important and novel area of investigation.

The present study aimed to compare histopathological characteristics of BCCs diagnosed via teledermatology versus face-to-face dermatology consultations in a tertiary hospital, with particular attention to tumor depth and histologic subtype.

## Materials and methods

### Study design

This retrospective observational study was conducted at a tertiary referral hospital in southern Europe. We compared two cohorts of patients with histologically confirmed BCC: those diagnosed in 2019 through face-to-face dermatology consultations (pre-pandemic period) and those diagnosed in 2022 via teledermatology referrals (post-pandemic period). Selecting these 2 years minimized the potential influence of healthcare disruptions caused by the COVID-19 pandemic on the observed differences. All patients were managed within the same tertiary care hospital system. Both face-to-face and teledermatology consultations were conducted by the same group of dermatologists, all of whom received standardized training for handling teleconsultations to minimize variability in diagnostic responses. In cases of uncertainty, the consultation was reviewed by a specialist dermatologic oncologist.

In the conventional in-person model, patients were referred by primary care physicians to outpatient dermatology clinics, with an average delay of 55.8 days between referral and evaluation. In contrast, the teleconsultation model employed a store-and-forward system in which primary care physicians submitted clinical images—when available, including dermoscopic images—for asynchronous review by dermatologists. Diagnostic feedback was provided within 24 h, and patients requiring in-person assessment were scheduled within 48 h of diagnosis. However, dermoscopic imaging capabilities were not uniformly available across all referring primary care centers.

#### Inclusion and exclusion criteria

Eligible participants were adults (≥18 years) with histologically confirmed basal cell carcinoma diagnosed through excisional biopsy. Patients diagnosed via punch or incisional biopsy, those who underwent surgical management at other institutions, or those with missing essential data were excluded. A total of 486 patients were included: 201 diagnosed through face-to-face consultations in 2019 and 285 diagnosed via teledermatology in 2022.

#### Study variables

Clinical and histopathologic data were extracted from electronic medical records (Diraya^®^ system) and pathology reports. Sociodemographic variables included patient age and sex. Clinical variables encompassed the diagnostic impression at first consultation (classified as BCC, non-BCC, or BCC among differential diagnoses), use of antiaggregant or anticoagulant therapy (none, antiaggregant, anticoagulant), consultation year (2019 vs. 2022), and consultation modality (face-to-face vs. teledermatology). Tumor-related variables included anatomical site (head and neck, trunk, upper extremities, lower extremities), presence of high-risk location (periocular, perinasal, perioral, or auricular), histologic subtype (superficial, nodular, micronodular, or sclerodermiform), and a combined histologic classification defined as non-aggressive (superficial or nodular) versus aggressive (micronodular or sclerodermiform). This binary classification reflects established clinical risk stratification frameworks used in the American Academy of Dermatology (AAD) Guidelines for the Management of Basal Cell Carcinoma (10) and in European dermato-oncology recommendations (3). These guidelines categorize superficial and nodular BCCs as low-risk growth patterns, while micronodular and sclerodermiform tumors are considered high-risk or aggressive due to their infiltrative behavior, subclinical extension, and higher recurrence risk. The regrouping therefore follows biologically meaningful and clinically actionable criteria. Pathological features included maximum diameter (DM), minimum diameter (dm), derived tumor diameters (DMxdm), tumor depth (mm), margin status, and the presence or absence of lymphovascular and perineural invasion.

High-risk BCC was defined according to international guidelines (10) as tumors located in the facial “H-zone”, hands, feet or genitalia; ≥20 mm on low-risk sites or ≥10 mm on intermediate-risk sites; recurrent tumors; lesions in previously irradiated areas; those occurring in immunosuppressed patients; or histologically aggressive subtypes (infiltrative, micronodular, morpheaform, basosquamous). Perineural invasion was also considered a high-risk feature.

#### Statistical analysis

Continuous variables were summarized as means and compared between groups using Welch’s two-sample *t*-test. Categorical variables were compared with Pearson’s chi-square test with Yates’ correction when appropriate. A multivariable logistic regression model was fitted to identify independent predictors of consultation modality (teledermatology vs. face-to-face). Significance was set at *p* < 0.05. Analyses were performed using R software (version 4.4.3), and group differences were visualized with the *ggplot2* package.

This study was conducted by collecting data from medical records and pathology reports, without any interventions that could pose a risk to patients. The study adhered to the ethical principles outlined in the Declaration of Helsinki.

## Results

### Patient characteristics

Of the 486 patients included, 201 (41.4%) were diagnosed through face-to-face consultations and 285 (58.6%) through teledermatology. Mean age was comparable between groups (71.8 vs. 70.9 years; *p* = 0.47), and sex distribution was similar (59.7% vs. 57.5% male; *p* = 0.68). Baseline demographic and clinical characteristics were balanced across pathways (Table 1).

**Table 1:** Baseline characteristics of patients with basal cell carcinoma, stratified by consultation pathway.

Variable	Face-to-face (*N* = 201)	Teledermatology (*N* = 285)	*p*-value
Age, mean (SD), years	71.8 (13.2)	70.9 (13.5)	0.47
Male sex, *n* (%)	120 (59.7)	164 (57.5)	0.68
Histologic subtype, *n* (%)			0.002
Superficial	32 (15.9)	25 (8.8)	
Nodular	125 (62.2)	187 (65.6)	
Micronodular	8 (4.0)	38 (13.3)	
Sclerodermiform	36 (17.9)	35 (12.3)	
Location, *n* (%)			0.08
Head and neck	142 (70.6)	223 (78.2)	
Trunk	41 (20.4)	40 (14.0)	
Upper extremities	11 (5.5)	12 (4.2)	
Lower extremities	7 (3.5)	10 (3.5)	
Positive margins, *n* (%)	8 (4.0)	11 (3.9)	

Values are presented as mean (standard deviation) or *n* (%). *p*-values were derived from Welch’s *t*-test (continuous variables) and Pearson’s chi-square test with Yates’ correction (categorical variables).

### Tumor depth and size

**Figure 1:**
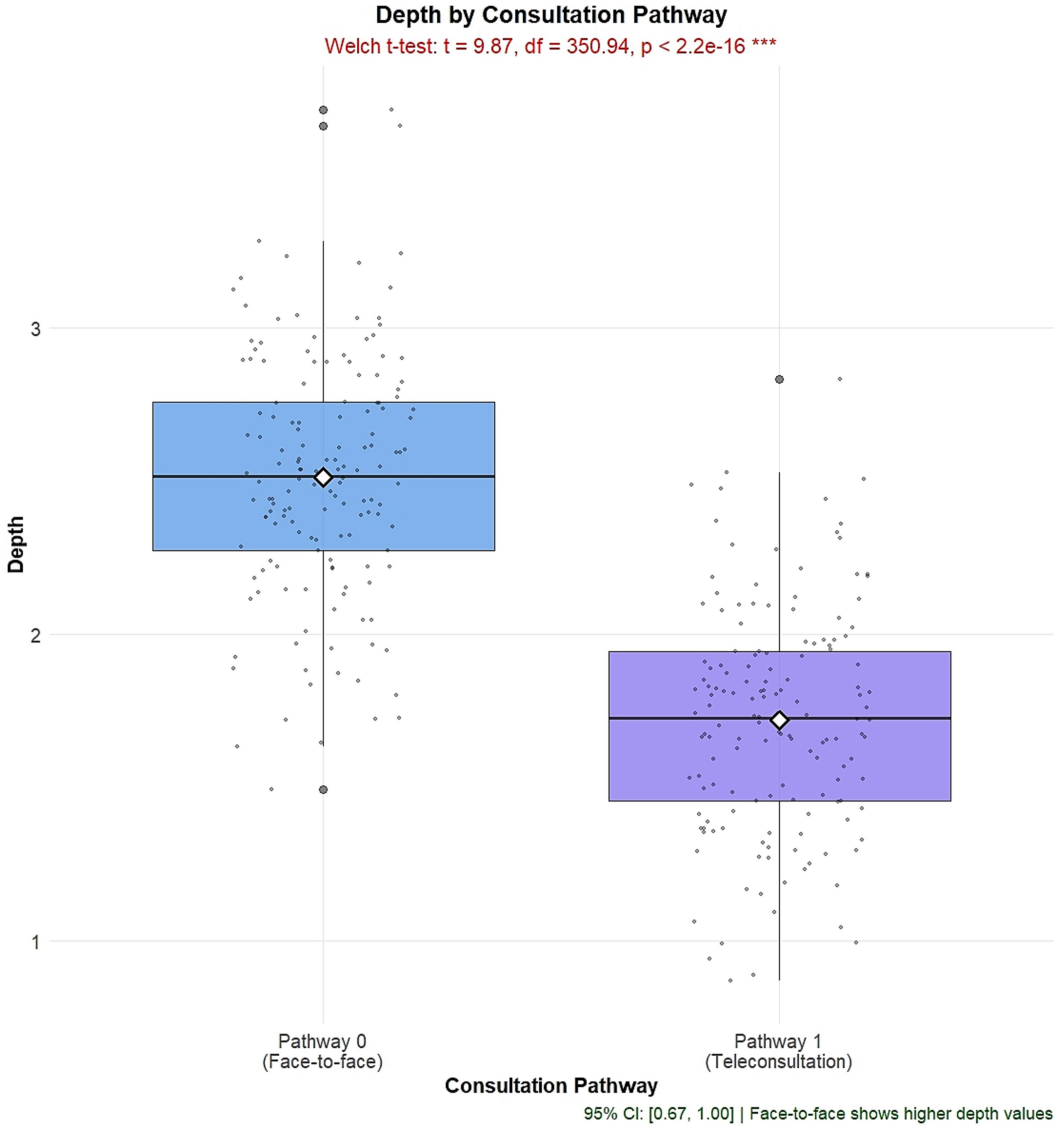
Tumor depth by consultation pathway. Boxplot comparing basal cell carcinoma depth between face-to-face consultations (2019) and teledermatology (2022). Mean depth was significantly lower in the teledermatology cohort (1.67 mm vs. 2.51 mm; Welch’s *t*-test, *t* = 9.87, df = 350.94, *p* < 0.001). Diamonds indicate mean values; whiskers denote the 95% confidence interval.

Mean tumor depth was significantly lower in teledermatology (1.67 mm) compared with face-to-face consultations (2.51 mm; Welch’s *t* = 9.87, *p* < 0.001) (Figure 1). Tumor diameter measurements (maximum, minimum, derived indices) did not show clinically meaningful differences.

### Histologic subtype

**Figure 2:**
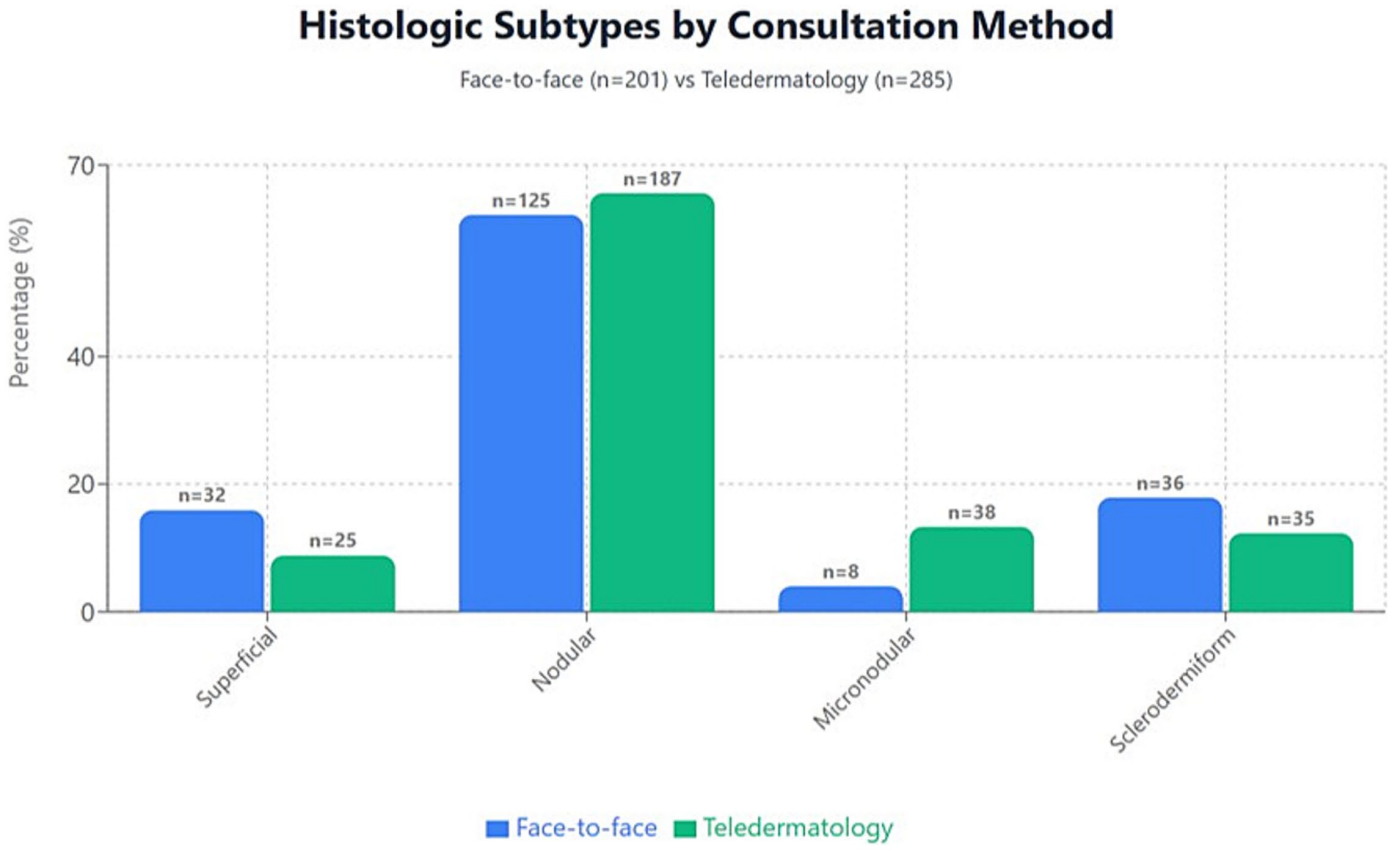
Distribution of histologic subtypes of basal cell carcinoma by consultation pathway. Bars show the frequency of superficial, nodular, micronodular, and sclerodermiform BCCs in face-to-face consultations (blue) and teledermatology (green). Superficial tumors were more frequent in face-to-face consultations (15.9% vs. 8.8%), while nodular and micronodular variants predominated in teledermatology (65.6% vs. 62.2 and 13.3% vs. 4.0%, respectively). Sclerodermiform tumors were slightly more frequent in face-to-face evaluations (17.9% vs. 12.3%). Frequencies correspond exactly to those reported in Table 1 (χ^2^ = 14.7; *p* = 0.002).

Histopathologic subtype distribution differed significantly between consultation pathways (χ^2^ = 14.7; *p* = 0.002). Superficial BCCs were more frequent in face-to-face consultations (15.9% vs. 8.8%), whereas nodular and micronodular subtypes predominated in the teledermatology cohort (65.6% vs. 62.2 and 13.3% vs. 4.0%, respectively). Sclerodermiform tumors were slightly more frequent in face-to-face evaluations (17.9% vs. 12.3%) (Figure 2).

When subtypes were regrouped into risk-based categories (non-aggressive: superficial/nodular; aggressive: micronodular/sclerodermiform), the distribution also differed between pathways (Supplementary Figure 1). However, this reclassification did not reveal a predominance of low-risk histology in the teledermatology cohort; instead, differences reflected the underlying subtype shifts described above, with teledermatology capturing more nodular and micronodular tumors while face-to-face consultations retained a higher proportion of superficial BCCs.

### Location and high-risk areas

Most lesions were located on the head and neck, with a higher proportion in the teledermatology group (78.2% vs. 70.6%). Trunk lesions accounted for 20.4% of face-to-face cases and 14.0% of teledermatology cases. The distribution across upper and lower extremities was similar between pathways (Table 1).

### Margin status and invasion

Positive surgical margins occurred at similar frequencies in both pathways (4.0% vs. 3.9%). Lymphovascular and perineural invasion were rare (<5%) and comparable between groups.

### Multivariable analysis

**Figure 3:**
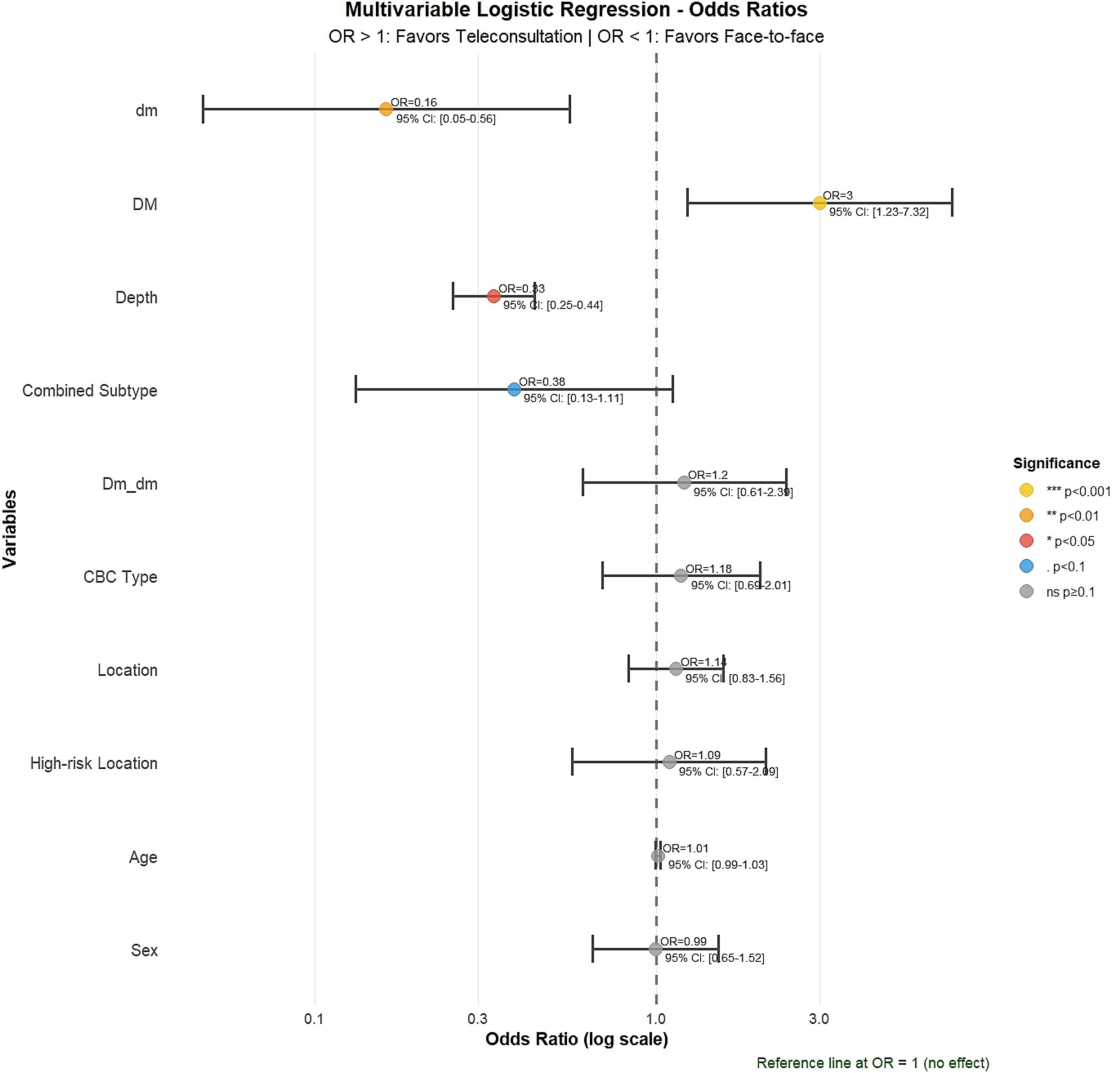
Multivariable logistic regression: odds ratios for predictors of consultation pathway. Forest plot showing odds ratios (95% CI) for predictors of teledermatology versus face-to-face consultation. Tumor depth was the only independent predictor (OR = 0.33, 95% CI: 0.25–0.44, *p* < 0.001), indicating that shallower tumors were strongly associated with teledermatology.

In logistic regression, tumor depth remained the only independent predictor of consultation modality (*β* = −1.10, *p* < 0.001) (Table 2; Figure 3). Age, sex, tumor site, high-risk location, diameter, margin status, and invasion variables were not significant predictors. The relative effect size and significance of predictors are summarized in a coefficient plot (Supplementary Figure 2). Although variables DM/dm reached statistical significance, they lack clear clinical interpretation, and tumor depth remained the only robust predictor of consultation pathway.

**Table 2:** Multivariable logistic regression for predictors of consultation pathway (teledermatology vs. face-to-face).

Variable	OR	95% CI	*p*-value
Sex (female vs. male)	1.00	0.66–1.53	0.99
Age (per year)	1.01	0.99–1.03	0.23
Histologic subtype (categorical)	1.18	0.69–2.02	0.54
Combined subtype (aggressive vs. non-aggressive)	0.38	0.13–1.11	0.08
Location (categorical)	1.13	0.83–1.56	0.44
High-risk site	1.09	0.57–2.09	0.80
DM	**3.48**	1.72–7.04	**0.0005**
dm	**0.21**	0.09–0.48	**0.0003**
Dm_dm	1.59	n.e. – n.e.	1.00
Tumor depth (per mm)	**0.33**	0.25–0.43	**<0.001**

OR, odds ratio; CI, confidence interval. Significant predictors (*p* < 0.05) are highlighted in bold.

## Discussion

In this retrospective cohort study, BCCs diagnosed through teledermatology exhibited significantly lower tumor depth and a higher prevalence of non-aggressive histologic subtypes compared with those diagnosed through face-to-face consultations. The mean depth was 1.67 mm in the teledermatology group versus 2.51 mm in the face-to-face group, yielding an absolute difference of 0.84 mm. Although less than 1 mm, this difference is clinically relevant, particularly in facial tumors, where even submillimetric variations in invasion depth can substantially increase the risk of incomplete excision, surgical complexity, and functional or cosmetic morbidity. To our knowledge, this is the first study to analyze histopathological features of BCC according to the diagnostic pathway. Previous studies on teledermatology have primarily focused on diagnostic concordance between remote clinical assessments and histopathological confirmation, consistently reporting moderate to high agreement rates, particularly when dermoscopy is utilized (7–9). Our results therefore provide novel evidence that teledermatology may not only be diagnostically accurate, but also associated with the detection of earlier-stage tumors.

Histologic subtype and tumor depth are critical prognostic factors in BCC. Nodular and superficial variants are generally considered non-aggressive, whereas micronodular, infiltrative, morpheaform, and basosquamous subtypes are associated with higher recurrence rates and greater potential for subclinical spread (1, 4, 11). *In our cohort,* individual subtype distribution differed significantly between pathways: nodular and micronodular tumors were more frequently diagnosed via teledermatology, whereas superficial BCCs were more common in face-to-face consultations, and sclerodermiform subtypes appeared at similar frequencies. When applying a simplified risk-based grouping, non-aggressive subtypes (superficial/nodular) were more frequent in the face-to-face cohort, while aggressive subtypes (micronodular/sclerodermiform) predominated in teledermatology.

This pattern is clinically coherent with the natural history of BCC. Nodular tumors—although classified as non-aggressive—are often the earliest morphologically apparent subtype, more likely to prompt patient attention and to be photographed and referred through teledermatology. In contrast, superficial BCCs, which frequently arise on the trunk, were more common in the face-to-face cohort, reflecting differences in anatomical distribution. Importantly, despite these subtype shifts, tumor depth remained significantly lower in the teledermatology cohort. Taken together, these findings indicate that teledermatology preferentially captures BCCs at an earlier histologic stage, even though its subtype distribution includes a higher proportion of micronodular tumors.

Tumor depth has also been shown to correlate strongly with surgical difficulty and risk of incomplete excision: lesions exceeding 6 mm are significantly more likely to present positive margins and require complex reconstruction (11, 12). The predominance of shallower lesions among teledermatology cases is therefore clinically relevant. Previous studies have reported that aggressive BCC variants exhibit higher cyclooxygenase-2 expression, which may contribute to their invasive biological behavior (13). Although our study did not assess this molecular pathway, these data provide a biological rationale for the unfavorable clinical course typically observed in infiltrative BCC subtypes.

In line with our results, previous evidence has highlighted the critical impact of diagnostic delay in BCC. Husein-ElAhmed et al. (14) reported a mean delay of nearly 20 months before diagnosis, with more than half of patients waiting over 1 year to seek medical attention (14). Older age, absence of personal or familial history of BCC, extra-facial location, and lack of alarming symptoms such as bleeding or pruritus were identified as key determinants of delay (14). Interestingly, their study did not find a significant association between diagnostic delay and tumor thickness, suggesting that biological growth patterns may modulate the effect of time to diagnosis. In our cohort, however, teledermatology was associated with significantly reduced tumor depth and a predominance of non-aggressive histologic subtypes, likely reflecting its ability to mitigate part of the structural delay inherent to patient- and system-dependent factors. Together, these findings underscore the potential of teledermatology to act not only as a diagnostic adjunct but also as a tool to reduce time-to-diagnosis in BCC, particularly in populations at risk for late presentation.

The clinical relevance of these findings extends beyond surgical outcomes. Locally advanced BCC (laBCC) represents a minority of cases but is associated with considerable morbidity, often necessitating extensive or mutilating surgery, or systemic therapy with Hedgehog pathway inhibitors (HHIs) (5, 6, 15–17). Although effective, vismodegib and sonidegib are associated with significant toxicity, development of resistance, and substantial financial costs. By enabling earlier detection of tumors with favorable histology, teledermatology may help reduce progression to laBCC and the subsequent need for systemic therapy. Furthermore, our findings support the role of teledermatology as a triage tool, providing timely referrals, shorter time-to-treatment, and improved access to dermatologic care, as highlighted by recent systematic reviews (9, 18, 19).

Several surgical series have confirmed that incomplete excision, and thereby risk of recurrence, is significantly associated with tumor depth, aggressive histology, and high-risk anatomical sites (11, 12). Clinical guidelines confirm that aggressive histologic subtypes, such as micronodular, infiltrative, and morpheaform BCC, are associated with greater risk of recurrence despite apparently adequate surgery (3, 10). Our study adds a novel perspective by demonstrating that these high-risk histologic features are more prevalent in tumors diagnosed through face-to-face consultations, whereas teledermatology preferentially detects early-stage disease. This finding aligns with prior reports indicating that teledermatology can improve time to biopsy and excision for keratinocyte carcinomas (9, 18, 19), supporting its role in timely cancer care.

Anatomical site is known to influence histologic subtype distribution, with superficial BCCs occurring more frequently on the trunk, as previously demonstrated (20). In our cohort, trunk lesions were indeed more frequent in the face-to-face group (20.4% vs. 14.0%), a pattern that likely contributes to the higher proportion of superficial BCCs observed in this pathway. However, head and neck lesions still predominated in both cohorts, and nodular and micronodular subtypes remained more frequent in the teledermatology group. These findings suggest that anatomical location may partially account for the subtype differences, but does not fully explain the distinct histologic profile and lower tumor depth observed in teledermatology cases. Clinical visibility and referral dynamics are likely additional contributors.

Beyond histopathologic differences, organizational metrics further underscore the value of teledermatology in tertiary care settings. At our institution, the structural implementation of teledermatology after the COVID-19 pandemic markedly reduced waiting times: the average interval from referral to dermatologic evaluation decreased from 55 days in the face-to-face model to just 2 days with teleconsultation, while mean delays for deferred surgical excision under local anesthesia fell from 16 to 7 days. These improvements, consistent with the clinical benchmarks outlined in the *Integrated Care Process for Skin Cancer* (Proceso Asistencial Integrado de Cáncer de Piel) established by the Andalusian Regional Health Ministry (Servicio Andaluz de Salud) (21), likely contribute to the detection of earlier-stage tumors by minimizing diagnostic and treatment delays. Such operational advantages complement the histopathologic findings of shallower depth and less aggressive subtypes in the teledermatology cohort, reinforcing its role as a structural tool to optimize oncologic outcomes and healthcare efficiency.

The main strengths of this study include its histologically confirmed dataset, relatively large sample size, and comparison of pre- and post-pandemic cohorts, which minimizes the confounding effect of COVID-19 on healthcare access. Limitations include its retrospective design, single-center scope, and the absence of follow-up data on recurrence or long-term outcomes. Additionally, unmeasured factors such as patient socioeconomic background or delay from symptom onset to consultation may have influenced tumor characteristics at presentation.

A potential referral bias may have influenced the histologic composition of the teledermatology cohort, as primary care physicians could have preferentially directed clinically less suspicious or early-stage lesions to remote assessment during the pandemic. Nevertheless, several lines of evidence suggest that this mechanism alone cannot account for the observed differences. Both cohorts were comparable in age, sex, and anatomic site distribution, indicating similar overall case profiles. Moreover, nodular and micronodular variants were more frequent in the teledermatology pathway, suggesting that remote consultation captured visible early-stage nodular tumors before deeper invasion occurred. Finally, in multivariable analysis, tumor depth remained the only independent predictor of consultation pathway (*β* = −1.10; *p* < 0.001), supporting the interpretation that teledermatology facilitates earlier histologic diagnosis beyond mere triage bias. Taken together, these findings reinforce the clinical utility of teledermatology not only as a triage system but also as an effective diagnostic gateway for timely identification of basal cell carcinoma at less advanced stages.

Future prospective, multicenter studies are warranted to confirm these findings and to evaluate whether the earlier detection associated with teledermatology translates into lower recurrence, reduced incomplete excision, and decreased incidence of laBCC requiring HHIs. The incorporation of teledermoscopy and artificial intelligence could further increase diagnostic sensitivity, particularly for aggressive histologic variants (22, 23). Moreover, dedicated studies in high-risk groups, such as immunosuppressed patients or those with multiple BCCs, would clarify whether teledermatology offers specific benefits in these populations (24–27).

## Conclusion

In conclusion, teledermatology was associated with the diagnosis of basal cell carcinomas with significantly lower tumor depth compared with those detected in face-to-face consultations. Although the distribution of histologic subtypes differed between pathways—with a higher proportion of superficial tumors in face-to-face care and a greater representation of nodular and micronodular variants in teledermatology—aggressive subtypes identified through teledermatology were nonetheless diagnosed at markedly shallower stages. These findings suggest that teledermatology facilitates earlier histologic detection across multiple BCC growth patterns, reinforcing its value as a structural tool to expedite assessment, minimize diagnostic delays, and potentially improve surgical and oncologic outcomes.

## Data Availability

The original contributions presented in the study are included in the article/Supplementary material, further inquiries can be directed to the corresponding author.

## Supplementary material

Supplementary Figure 1: Distribution of regrouped histologic subtypes (non-aggressive vs aggressive) by consultation pathway.

Supplementary Figure 2: Coefficient/summary plot of multivariable model predictors (effect sizes and significance).

## References

[1] Kim DP Kus KJB Ruiz E. Basal Cell Carcinoma Review. Hematol Oncol Clin North Am. (2019) 33:13–24. doi: 10.1016/j.hoc.2018.09.004, 30497670

[2] Lomas A Leonardi-Bee J Bath-Hextall F. A systematic review of worldwide incidence of nonmelanoma skin cancer. Br J Dermatol. (2012) 166:1069–80. doi: 10.1111/j.1365-2133.2012.10830.x, 22251204

[3] Peris K Fargnoli MC Kaufmann R Arenberger P Bastholt L Seguin NB . European consensus-based interdisciplinary guideline for diagnosis and treatment of basal cell carcinoma-update 2023. Eur J Cancer. (2023) 192:113254. doi: 10.1016/j.ejca.2023.113254, 37604067

[4] Cameron MC Lee E Hibler BP Barker CA Mori S Cordova M . Basal cell carcinoma: epidemiology; pathophysiology; clinical and histological subtypes; and disease associations. J Am Acad Dermatol. (2019) 80:303–17. doi: 10.1016/j.jaad.2018.03.060, 29782900

[5] Sekulic A Migden MR Basset-Seguin N Garbe C Gesierich A Lao CD . Long-term safety and efficacy of vismodegib in patients with advanced basal cell carcinoma: final update of the pivotal ERIVANCE BCC study. BMC Cancer. (2017) 17:332. doi: 10.1186/s12885-017-3286-5, 28511673 PMC5433030

[6] Dummer R Lear JT Guminski A Leow LJ Squittieri N Migden M. Efficacy of sonidegib in histologic subtypes of advanced basal cell carcinoma: results from the final analysis of the randomized phase 2 basal cell carcinoma outcomes with LDE225 treatment (BOLT) trial at 42 months. J Am Acad Dermatol. (2021) 84:1162–4. doi: 10.1016/j.jaad.2020.08.042, 33358380

[7] Finnane A Dallest K Janda M Soyer HP. Teledermatology for the diagnosis and Management of Skin Cancer: a systematic review. JAMA Dermatol. (2017) 153:319–27. doi: 10.1001/jamadermatol.2016.4361, 27926766

[8] Tensen E van der Heijden JP Jaspers MWM Witkamp L. Two decades of teledermatology: current status and integration in national healthcare systems. Curr Dermatol Rep. (2016) 5:96–104. doi: 10.1007/s13671-016-0136-7, 27182461 PMC4848332

[9] Nikolakis G Vaiopoulos AG Georgopoulos I Papakonstantinou E Gaitanis G Zouboulis CC. Insights, advantages, and barriers of teledermatology vs. face-to-face dermatology for the diagnosis and follow-up of non-melanoma skin cancer: a systematic review. Cancer. (2024) 16:578. doi: 10.3390/cancers16030578, 38339329 PMC10854718

[10] Kim JYS Kozlow JH Mittal B Moyer J Bichakjian C Armstrong A . Guidelines of care for the management of basal cell carcinoma. J Am Acad Dermatol. (2018) 78:540–59. doi: 10.1016/j.jaad.2017.10.006, 29331385

[11] Breuninger H Dietz K. Prediction of subclinical tumor infiltration in basal cell carcinoma. J Dermatol Surg Oncol. (1991) 17:574–8. doi: 10.1111/j.1524-4725.1991.tb03655.x, 1860987

[12] Husein-Elahmed H Aneiros-Fernandez J Gutierrez-Salmeron MT Aneiros-Cachaza J Naranjo-Sintes R. Basal cell carcinoma: analysis of factors associated with incomplete excision at a referral hospital in southern Spain. Cutis. (2014) 93:155–61. 24738098

[13] Husein-El Ahmed H Aneiros-Fernandez J Gutierrez-Salmeron MT Aneiros-Cachaza J Naranjo-Sintes R. Effect of non-steroidal anti-inflammatory drugs on the histology of basal cell carcinomas. Eur J Dermatol. (2012) 22:205–10. doi: 10.1684/ejd.2011.1625, 22240452

[14] Husein-Elahmed H Gutierrez-Salmeron MT Naranjo-Sintes R Aneiros-Cachaza J. Factors related to delay in the diagnosis of basal cell carcinoma. J Cutan Med Surg. (2013) 17:27–32. doi: 10.2310/7750.2012.12030, 23364147

[15] Epstein EH. Basal cell carcinomas: attack of the hedgehog. Nat Rev Cancer. (2008) 8:743–54. doi: 10.1038/nrc2503, 18813320 PMC4457317

[16] Fecher LA. Systemic therapy for inoperable and metastatic basal cell cancer. Curr Treat Options in Oncol. (2013) 14:237–48. doi: 10.1007/s11864-013-0233-9, 23558911

[17] Migden MR Guminski A Gutzmer R Dirix L Lewis KD Combemale P . Treatment with two different doses of sonidegib in patients with locally advanced or metastatic basal cell carcinoma (BOLT): a multicentre, randomised, double-blind phase 2 trial. Lancet Oncol. (2015) 16:716–28. doi: 10.1016/S1470-2045(15)70100-2, 25981810

[18] Morton CA Downie F Auld S Smith B van der Pol M Baughan P . Community photo-triage for skin cancer referrals: an aid to service delivery. Clin Exp Dermatol. (2011) 36:248–54. doi: 10.1111/j.1365-2230.2010.03960.x, 21070338

[19] Naka F Lu J Porto A Villagra J Wu ZH Anderson D. Impact of dermatology eConsults on access to care and skin cancer screening in underserved populations: a model for teledermatology services in community health centers. J Am Acad Dermatol. (2018) 78:293–302. doi: 10.1016/j.jaad.2017.09.017, 29061478

[20] Ghanadan A Abdollahi P Rabet M Naraghi Z Abbasi MA Moslehi H . Different anatomical distribution of basal cell carcinoma subtypes in Iranian population: association between site and subtype. Ann Dermatol. (2014) 26:559–63. doi: 10.5021/ad.2014.26.5.559, 25324646 PMC4198581

[21] Junta de Andalucía. Proceso asistencial integrado cáncer de piel, 2a ed. (2014). Available online at: https://www.juntadeandalucia.es/organismos/saludyconsumo/areas/calidad/pai/paginas/pai-cancer-piel.html (accessed June 15, 2025).

[22] Börve A Dahlén Gyllencreutz J Terstappen K Johansson Backman E Aldenbratt A Danielsson M . Smartphone teledermoscopy referrals: a novel process for improved triage of skin cancer patients. Acta Derm Venereol. (2015) 95:186–90. doi: 10.2340/00015555-1906, 24923283

[23] Tan E Yung A Jameson M Oakley A Rademaker M. Successful triage of patients referred to a skin lesion clinic using teledermoscopy (IMAGE IT trial). Br J Dermatol. (2010) 162:803–11. doi: 10.1111/j.1365-2133.2010.09673.x, 20222920

[24] Garrett GL Blanc PD Boscardin J Lloyd AA Ahmed RL Anthony T . Incidence of and risk factors for skin Cancer in organ transplant recipients in the United States. JAMA Dermatol. (2017) 153:296–303. doi: 10.1001/jamadermatol.2016.4920, 28097368

[25] O’Reilly Zwald F Brown M. Skin cancer in solid organ transplant recipients: advances in therapy and management: part I. Epidemiology of skin cancer in solid organ transplant recipients. J Am Acad Dermatol. (2011) 65:253–61. doi: 10.1016/j.jaad.2010.11.062 21763561

[26] Euvrard S Kanitakis J Claudy A. Skin cancers after organ transplantation. N Engl J Med. (2003) 348:1681–91. doi: 10.1056/nejmra022137, 12711744

[27] Bouwes Bavinck JN Euvrard S Naldi L Nindl I Proby CM Neale R . Keratotic skin lesions and other risk factors are associated with skin cancer in organ-transplant recipients: a case-control study in the Netherlands, United Kingdom, Germany, France, and Italy. J Invest Dermatol. (2007) 127:1647–56. doi: 10.1038/sj.jid.5700776, 17380113 PMC2478722

