# Supplementary figures and images for: Teledermatology and face-to-face pathways for basal cell carcinoma diagnosis in a southern European cohort: a comparative histopathologic analysis
Source: Front Med (Lausanne). 2026 Feb 2;13:1713904. doi: 10.3389/fmed.2026.1713904 (PMC12907131; doi:10.3389/fmed.2026.1713904)

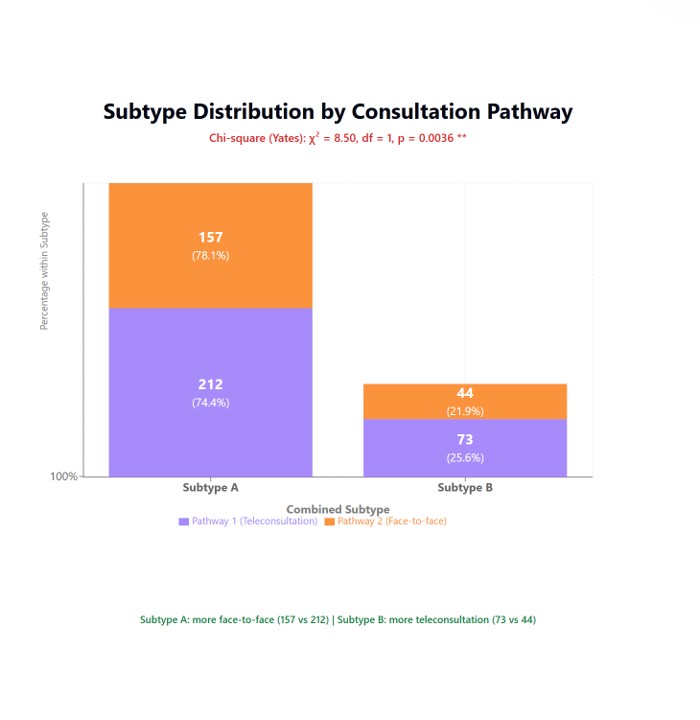

Supplement: Supplementary Figure 1: — Distribution of regrouped histologic subtypes (non-aggressive vs aggressive) by consultation pathway. [file Image_1.JPEG]

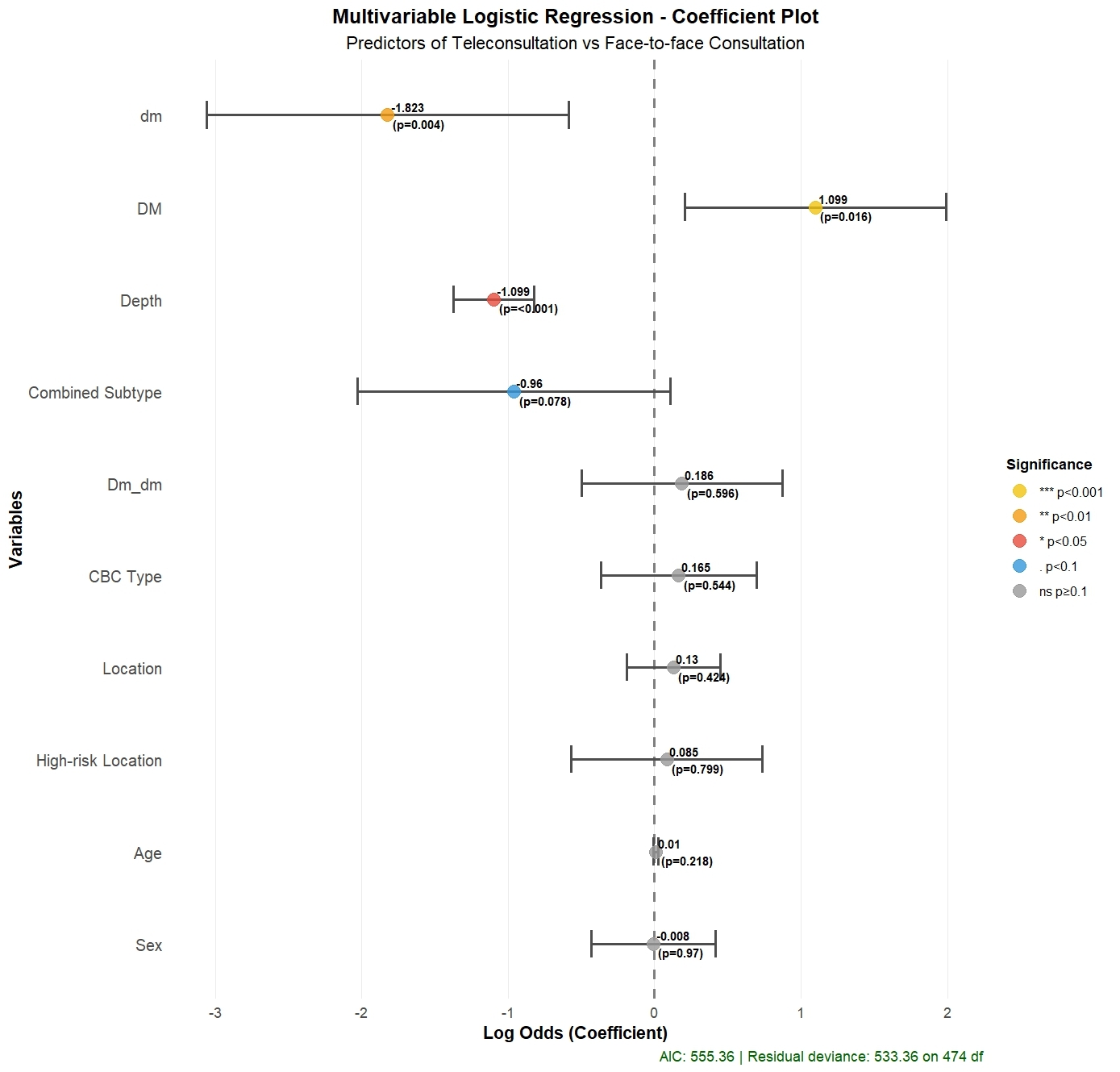

Supplement: Supplementary Figure 2: — Coefficient/summary plot of multivariable model predictors (effect sizes and significance). [file Image_2.JPEG]
